# The Use of Epidermal Growth Factor Receptor Monoclonal Antibodies in Squamous Cell Carcinoma of the Head and Neck

**DOI:** 10.1155/2012/761518

**Published:** 2012-09-13

**Authors:** Jeffery S. Russell, A. Dimitrios Colevas

**Affiliations:** Division of Oncology, Department of Medicine, Stanford University Medical Center, Stanford University, Stanford, CA 94305, USA

## Abstract

Targeting of the EGF receptor (EGFR) has become a standard of care in several tumor types. In squamous cell carcinoma of the head and neck, monoclonal antibodies directed against EGFR have become a regular component of therapy for curative as well as palliative treatment strategies. These agents have anti-tumor efficacy as a single modality and have demonstrated synergistic tumor killing when combined with radiation and/or chemotherapy. While cetuximab has been the primary anti-EGFR monoclonal antibody used in the US, variant anti-EGFR monoclonal antibodies have been used in several clinical studies and shown benefit with improved toxicity profiles. Next generation anti-EGFR monoclonal antibodies may demonstrate multi-target epitope recognition, enhanced immune cell stimulation, or conjugation with radioisotopes in order to improve clinical outcomes. Identification of the specific patient subset that would optimally benefit from anti-EGFR monoclonal antibodies remains an elusive goal.

## 1. Introduction

In 2012, head and neck cancers of the oral cavity and pharynx will make up an estimated 2.5% of cancer diagnoses in the United States and for the 40,250 new cases diagnosed, there will be an estimated 7,850 deaths [1]. Worldwide, head and neck cancers are approximately 5% of all new cancer diagnoses, with a large proportion of these cases originating in developing countries [2]. Locally advanced squamous cell carcinoma of the head and neck (SCCHN) has cure rates of only 30–60%, even with combined therapeutic approaches [3]. Local recurrence rates of 30–50% and distant metastasis rates of 13–22% illustrate the need for more effective therapies [4, 5].

Towards this end, molecular analysis of SCCHN has found the overexpression of the epidermal growth factor receptor (EGFR) at rates of up to 90% in tumors and EGFR overexpression has been associated with a poor prognosis [6–11]. The deregulation or inappropriate activation of the EGFR family members has been shown to drive oncogenic transformation, tumor cell proliferation, and cell survival pathways in a variety of malignancies [12–14]. Ligand binding or mutations within the EGF receptor cause activation of downstream signaling pathways, such as Ras/Raf/MAPK and PI3 K/Akt [15–17].

Thus, agents that specifically target EGFR and consequently its downstream signaling pathways are appealing candidates to enhance tumor cell killing, especially in high-expressing tumors such as SCCHN. Currently, therapy for targeting EGFR can be divided between small molecule tyrosine kinase inhibitors and monoclonal antibodies. In this paper, we will address the benefits of select monoclonal antibodies as anti-EGFR therapy in SCCHN (Table 1). This paper will focus on both curative as well as palliative treatment strategies. Furthermore, we aim to discuss treatment responses that have been enhanced with anti-EGFR monoclonal antibody therapy in combination with chemotherapy and/or radiation therapy. Finally, we will discuss novel approaches under development to improve the antitumor properties of EGFR directed monoclonal antibodies.

## 2. Early Development of Anti-EGFR Monoclonal Antibodies

Cetuximab (Erbitux; formerly IMC-C225) was the first monoclonal antibody used clinically to target the EGF receptor. It is a chimeric IgG1 antibody derived from both mouse and human immunoglobulin genes [40]. Cetuximab is specific for the EGFR/Her1receptor, does not cross-react with other Her receptor family members, and targets the extracellular EGFR domain [41, 42]. Cetuximab binds with a higher affinity than the native EGF ligand to modulate ligand-mediated dimerization and activation of the receptor [43]. In addition to blocking downstream EGFR signaling pathways crucial for tumor survival, cetuximab also stimulates antibody-dependent cellular cytotoxicity (ADCC) by recruiting activated immune cells into tumors to augment tumor cell killing [44–46].

Original work by Masui et al. demonstrated that anti-EGFR monoclonal antibodies were able to inhibit the growth of human tumor xenografts in nude mice when given at the time of tumor implantation [47]. *In vitro*, the antibodies demonstrated a primarily cytostatic effect against tumor cell lines, while *in vivo*, treatments with anti-EGFR antibodies were unable to prevent the growth of established tumors in xenograft models [41, 47]. Limitations in the *in vivo* setting may have been an inability of the antibody to penetrate into the core of the artificially placed tumors as well as the immunologic consequences using an immunodeficient mouse model (i.e., failure to fully activate the ADCC response). Additional preclinical work determined that an anti-EGFR monoclonal antibody added to cisplatin therapy significantly enhanced xenograft growth inhibition [48]. Several investigators also found that the addition of an EGFR monoclonal antibody enhanced radiation sensitivity of head and neck cell lines *in vitro*, as well as in nude mouse xenografts [18, 49, 50]. The radiation response of A431 tumor xenografts was enhanced with EGFR monoclonal antibody therapy and this response was noted to be maintained with continued antibody treatment after radiation [51, 52]. More recently, Zhang et al. combined cetuximab and radiation therapy with the addition of cisplatin chemotherapy which resulted in the decreased survival of several human head and neck cancer cell lines [53].

In 1991, the first phase I trial of a mouse monoclonal antibody (IgG1) against EGFR was completed in advanced lung cancer patients and demonstrated antibody uptake at the tumor site [54]. Similarly, Perez-Soler et al., using an IgG2 mouse monoclonal antibody, and Modjtahedi et al., using an IgG2 rat monoclonal antibody, targeted EGFR in patients with lung cancer and squamous cell carcinoma of the head and neck without significant toxicity [55, 56]. The tolerability of the early mouse monoclonal antibody studies along with the positive results from the preclinical combinations of chimeric anti-EGFR antibodies with chemotherapy and radiotherapy lead to the development of single agent as well as combinational phase I studies with cetuximab in humans [24, 57, 58]. These studies demonstrated that patients were able to tolerate the combination of anti-EGFR antibodies alone or in combination with either chemotherapy or radiation therapy. Only mild to moderate adverse events were noted with the most common adverse reaction being an acneiform rash.

## 3. Cetuximab: Curative Treatment

Nonsurgical curative treatment of locally advanced squamous cell carcinoma of the head and neck includes chemoradiation using concurrent platinum-based therapy as the most widely accepted standard of care. The benefit of adding cisplatin is often limited by toxicities or may not be feasible due to patient comorbidities such as diminished renal function, preexisting neuropathy, and poor auditory function. The clinical tolerability of cetuximab along with the preclinical data demonstrating benefit of adding cetuximab to radiation *in vitro* and *in vivo* led investigators to explore the use of cetuximab combined with radiation therapy in the curative setting (Table 2). Bonner et al. demonstrated in a phase III trial of 424 SCCHN patients randomized to radiation therapy alone or cetuximab and radiation therapy that the addition of cetuximab to radiation therapy increased the duration of locoregional control compared to radiation alone (24.4 months versus 14.9, HR 0.68, 95% CI 0.52–0.89, *P* = 0.005) [18]. Overall survival at 3 years also favored the cetuximab cohort (55% versus 45%, *P* = 0.05). The cetuximab and radiation arm had higher rates of rashes/skin reactions and infusion reactions; otherwise, there was no difference in grade 3/4 adverse events between treatment arms. This definitive study resulted in the FDA approving cetuximab for use in combination with radiation for the treatment of locally advanced squamous cell carcinoma of the head and neck in 2006. A follow-up report found that the overall 5 yr survival was 45.6% versus 36.4% in the cetuximab and radiation arm versus the radiation alone arm, respectively [19]. Their data also suggested improved overall survival in patients experiencing at least a grade 2 acneiform rash during treatment.

With positive outcomes supporting the combination of cetuximab and radiation, investigators began to determine if cetuximab could be used in combination with standard platinum-based chemoradiation regimens. Pfister et al. undertook a phase II study of 22 patients with locally advanced SCCHN using weekly cetuximab with radiation combined with two high-dose cisplatin treatments [20]. Toxicities included mucositis, nausea/vomiting, dehydration, and abnormal renal function along with the known cetuximab-related grade 3/4 toxicities of acneiform rash (10%) and infusion reactions (5%). Due to high levels of toxicity, the trial was stopped early; however, long-term follow-up of the participants found a 3 yr survival of 76%, 3 yr progression-free survival rates of 56%, and 3 yr locoregional control rates of 71%. Langer et al. reported using cisplatin (3 cycles) and weekly cetuximab concurrently with radiation therapy in 61 patients with locally advanced, unresectable SCCHN patients [21]. Objective responses were seen, but 97% of patients experienced a grade 3 or higher toxicity resulting in the observation that chemoradiation with cisplatin and cetuximab, while feasible, should be reserved for fit patients. An attempt to reduce toxicity was considered by Merlano et al. in a phase II study who used 3 cycles of low-dose cisplatin (20 mg/m^2^) given over 5 days with 5-FU in combination with weekly cetuximab and alternating weeks of daily XRT [22]. Median progression-free survival of over 21 months and overall survival of 32.6 months was reported. With regard to toxicity, all 45 patients in the trial experienced mucositis (36% grade 4) and radiodermatitis. Additionally, nearly 50% of patients required total parenteral nutrition support. Thus, toxicities are often the limiting factor in potentially beneficial regimens combining cetuximab with chemotherapy and radiation.

In 2011, results of the RTOG 0522 Phase III trial comparing cisplatin (given as 2 doses q 3 week) and radiation with and without weekly cetuximab in 895 locally advanced SCCHN patients were reported [23]. There was no difference in either progression-free survival (HR 1.05, 0.84–1.29; *P* = 0.66) or overall survival (HR 0.87, 0.66–1.15; *P* = 0.17) at two-year followup, despite increased toxicity, including mucositis and skin reactions in the cetuximab arm.

Questions remain if cetuximab combined with radiation is superior to cisplatin-based chemoradiotherapy. Caudell et al. retrospectively reviewed 31 patients treated with cetuximab and radiation and 103 patients with platinum-based chemotherapy and radiation and, after multivariate analysis, found no difference in 3 yr locoregional control rates and 3 yr overall survival between the treatment arms [59]. In contrast, a retrospective study from Koutcher et al. reviewed 174 locally advanced SCCHN patients treated with either cisplatin/XRT or cetuximab/XRT [60]. Results demonstrated 2 yr overall survival of 66.6% versus 44.5%, 2 yr progression-free survival of 92.8% versus 87.4%, and 2 yr local control failure rates of 5.7% versus 39.9%, in cisplatin/XRT combinations versus cetuximab/XRT combinations, respectively. Grade 3/4 toxicities were similar between treatment arms. Recently, Lefebvre et al. reported results from 153 patients with untreated, but surgically resectable, stage III/IV larynx or hypopharynx SCCHN who were randomized to treatment with either cisplatin + XRT or cetuximab + XRT following induction chemotherapy and found similar rates of laryngeal preservation (86% versus 82%) and survival (85% versus 86%) between the treatments with the cisplatin + XRT treatment arm experiencing higher rates of late toxicity [61]. An on-going phase III trial (RTOG 1016; NCT01302834) is comparing radiation therapy with cisplatin versus cetuximab in locally advanced SCCHN with 5 yr overall survival as the primary endpoint to determine which agent may be the most efficacious in patients with p16 positive oropharyngeal tumors.

## 4. Cetuximab: Palliative Treatment

Initial studies were designed to evaluate cetuximab in SCCHN patients with recurrent or metastatic disease (Table 3). In the trials discussed above, several patients demonstrated stable disease with cetuximab treatment alone [24]. Vermorken et al. conducted a phase II trial (*n* = 103) in recurrent or metastatic stage III/IV SCCHN patients with progressive disease on platinum-based therapy where patients received weekly infusions of cetuximab alone until disease progression [25]. Analysis of the intent-to-treat population demonstrated a single agent disease control rate of 13% (CR + PR + SD) while a subgroup that received platinum-based therapy with cetuximab showed disease control rates of 46%. Most common toxicities were grade 1 or 2 acne-like skin reactions. One death from an infusion-related reaction to cetuximab was reported. 

In the palliative setting, two phase II trials demonstrated the benefit of combining cetuximab with platinum-based therapy in patients with recurrent or metastatic SCCHN who had progressed on prior treatment. Baselga et al. (*n* = 96) found an overall response rate of 10% using combined platinum-based therapy and cetuximab in a population with prior platinum exposure [26]. Disease control rates (CD + PR + SD) were 53% and a median survival of 183 days was reported in the intent-to-treat group. Sixty percent developed skin reactions, typically an acneiform rash, but less than 15% had a grade 3 or 4 skin reaction. Three of ninety-six patients had cetuximab-related infusion reactions. Herbst et al. enrolled 132 patients with stable or progressive disease after platinum-based therapy to a trial of cetuximab combined with cisplatin [27]. The stable disease group exhibited an objective response rate of 18% and a disease control rate of 76%, while the progressive disease group (cohort 1) had rates of 20% and 64%, respectively. A second group with progression on prior treatment, with a longer delay before starting the experimental arm, demonstrated objective response rates of 6% and disease control rates of 52%. Infusion reactions were noted in approximately 13% of patients and the most common adverse event was an acne-like rash occurring in 70% of patients. In 2006, the FDA approved cetuximab as a single agent for the treatment of patients with recurrent or metastatic SCCHN for whom prior platinum-based therapy had failed.

A phase III trial designed for patients with recurrent or metastatic head and neck cancer without prior palliative treatment (*n* = 117) compared the use of cetuximab and cisplatin versus cisplatin alone [28]. The primary endpoint was progression-free survival; however, PFS was not significantly different between the treatment arms (4.2 months versus 2.7 months, *P* = 0.09, HR 0.78, 95% CI 0.54–1.12). Similar results were found for overall survival (9.2 months versus 8.0 months, *P* = 0.21); however, when survival was evaluated on the basis of time to develop skin toxicity, the estimated hazard ratio was 0.42 (95% CI 0.21–0.86) and *P* = 0.01. The objective response rate for the combinational therapy arm was 26% versus 10% in the cisplatin control group. Grade 3 and 4 toxicities were more frequent in the cetuximab and cisplatin cohort (90% versus 73%). Skin rash occurred in 77% of the patients on the cetuximab and cisplatin arm versus 24% in the cisplatin alone group.

Further supportive evidence for the use of cetuximab with chemotherapy in the palliative setting was seen in a phase I/II trial (*n* = 53) of cetuximab combined with either carboplatin or cisplatin and 5-FU, which demonstrated an overall response rate of 36% [29]. Adverse events were common, but only 15% of patients experienced a grade 3 or 4 adverse event. Acne-like rash was common in 56% of the treatment cohort.

These studies demonstrated that cetuximab could be used with cisplatin/5-FU treatments with modest, but tolerable, increases in adverse events and that the combination of cetuximab and cisplatin had improved response rates over chemotherapy alone. In 2008, Vermorken et al. investigated the use of cetuximab/platinum/5-FU versus platinum/5-FU as the first line treatment of patients with recurrent and metastatic SCCHN [30]. This phase III study evaluated 442 patients randomized to cetuximab + platinum-therapy/5-FU versus chemotherapy alone with a primary endpoint of overall survival. Treatment could be with either cisplatin or carboplatin at the discretion of the treating physician. Patients received up to 6 cycles of chemotherapy and responding patients remained on cetuximab alone until progression. In the cetuximab group, overall survival was 10.1 months versus 7.4 months in the chemotherapy alone group (HR 0.80; 95% CI 0.64–0.99; *P* = 0.04). Progression free survival was 5.6 months in the cetuximab group versus 3.3 months in the chemotherapy alone group (HR 0.54; 95% CI 0.43–0.67, *P* < 0.001). The incidence of grade 3 and 4 adverse events was similar between study groups; however, the cetuximab group had more cases of sepsis, hypomagnesemia, skin reactions, and infusion reactions compared to the chemotherapy alone group. This study resulted in the FDA, in November 2011, approving cetuximab in combination with platinum-based therapy plus 5-FU for the first-line treatment of patients with recurrent locoregional disease and/or metastatic squamous cell carcinoma of the head and neck.

Also in the palliative setting, cetuximab has been shown to be active in combination with other chemotherapy agents. Hitt et al. investigated the use of cetuximab with weekly paclitaxel as a first-line treatment for recurrent and/or metastatic disease and found an overall response rate of 54% and disease control rates (CR + PR + SD) of 80% with a median survival of 8.1 months [62]. Additionally, several small trials have demonstrated effective disease control using cetuximab in combination with taxanes and platinum-based therapy as first- and second-line interventions for recurrent and metastatic SCCHN [63–65]. Argiris et al. evaluated a combination of cetuximab with bevacizumab, an anti-VEGF monoclonal antibody, and noted partial response rates of 18% and stable disease 55% in heavily pretreated patients (*n* = 45). The median progression-free survival and overall survival were 2.8 and 7.6 months, respectively. Of note, the study generated 25 grade 4 adverse events and three grade 4 events [66]. Finally, a recent abstract found the combination of bendamustine and cetuximab in relapsed squamous cell carcinoma of the head and neck resulted in partial response rate of 36% and stable disease in 36% of patients (*n* = 28) who had failed prior therapy [67].

## 5. Cetuximab Summary

For treatment of SCCHN with curative intent, cetuximab combined with radiation therapy clearly has a benefit over radiation therapy alone. However, the addition of cetuximab to standard chemoradiation schemes using platinum-based therapy does not appear to enhance tumor response beyond that of standard chemoradiation, which remains the standard of care. As mentioned above, it is unknown if cetuximab is superior, equal to, or inferior to cisplatin-based chemoradiation, but this question will be pursued by future clinical trials. Questions also remain as to the benefit of cetuximab in combination with other chemotherapy agents and radiation as well as in other treatment regimens including induction chemotherapy and/or maintenance therapy. With respect to treatment of recurrent and/or metastatic SCCHN, the addition of cetuximab to platinum-based therapy and 5-FU improves patient survival and has recently been approved as first line therapy in the US. The above studies demonstrate clinical improvement with cetuximab, but there are also concerns for increased toxicity when combined with chemotherapy and/or radiation.

## 6. Alternative Anti-EGFR Monoclonal**** Antibodies (Table 4)

*Panitumumab* (Vectibix, formerly ABX-EGF) was the first fully humanized monoclonal IgG2 antibody developed against EGFR. Development of an IgG2 antibody resulted in an immunoglobulin species that is more resistant to enzyme degradation and consequently has a longer half-life [68, 69]. Additionally, the humanized IgG2 antibody was also designed to be less immunogenic and to reduce the risk of hypersensitivity reactions; however, there is a question as to whether an IgG2 monoclonal antibody is effective in recruiting ADCC for an immunologically mediated anticancer effect [70].

The SPECTRUM trial was a phase III study to evaluate panitumumab in combination with chemotherapy versus chemotherapy alone as first line therapy for metastatic and/or recurrent squamous cell carcinoma of the head and neck [37]. The trial enrolled 657 patients with recurrent or metastatic squamous cell carcinoma of the head and neck who were randomized to platinum-based (cisplatin or carboplatin) chemotherapy + 5-FU or panitumumab in combination with platinum/5-FU therapy and measured overall survival as the primary endpoint. Cycles could be repeated up to six times and patients on the panitumumab arm had the option of receiving maintenance therapy until toxicity or disease progression. Median survival in the panitumumab arm was 11.1 months compared to 9.0 months in the chemotherapy alone arm; however, this difference was not statistically significant (HR = 0.87, 95% CI: 0.73–1.05; *P* = 0.14). The safety profile was similar between treatment arms. Initial subgroup analysis of performance status, age, sex, prior radiotherapy, region, or site of disease found no significant difference between the cohorts. However, when overall response was assessed by RECIST criteria, the panitumumab containing arm demonstrated higher rates of partial responses (35 versus 24%) and better disease control (CR + PR+ SD; 84 versus 72%, *P* = 0.004). 

Further subgroup analysis of the SPECTRUM trial found that in an HPV-negative population subset, panitumumab-treated patients demonstrated overall survival benefit of 11.6 months versus 8.6 months (HR 0.73, *P* = 0.02); however, this trend was not present in an HPV-positive subset (HR 0.96, *P* = 0.88) [71]. The potential benefit to HPV-negative populations is intriguing, but it should be noted that this analysis was performed retrospectively and samples were from the primary tumor and not the recurrences.

Additional trials have combined panitumumab with chemotherapy and radiation to augment tumor response. Wirth et al. investigated escalating doses of paclitaxel given with panitumumab, carboplatin, and radiation therapy for previously untreated, advanced stage SCCHN [35]. This regimen was associated with moderate toxicity; however, for all patients that completed therapy, an objective response of over 95% was demonstrated. 

*Zalutumumab* (formerly HuMax-EGFr) is also a fully humanized anti-EGFR monoclonal antibody, an IgG1 isotype [72]. In a phase III trial (*n* = 286) with zalutumumab versus basic supportive care with optional methotrexate in platinum-refractory recurrent/metastatic SCCHN patients, Machiels et al. reported no significant difference in overall survival (6.7 months for zalutumumab arm and 5.2 months for control arm, HR 0.77 (97.06% CI 0.57–1.05), *P* = 0.065); however, progression-free survival was longer in the zalutumumab arm versus controls (9.9 weeks versus 8.4 weeks, HR 0.63 (95% CI 0.47–0.84), *P* = 0.0012). Overall the tumor response rate was 6.3% in the zalutumumab arm [38]. The safety profile was similar as the other anti-EFGR monoclonal antibodies with a grade 3-4 rash occurring most frequently (21%), although severe infusion-related reactions only occurred in 4% of patients. 

*Nimotuzumab* (formerly h-R3 mAb) is a humanized antibody (approximately 10% murine genes) with intermediate affinity to the EGF receptor. It was designed as an IgG1 isotype to enhance ADCC and potentiate tumor suppression, as well as to contain fewer foreign antigens to reduce infusion-related reactions [73]. A phase I trial revealed the antibody was well tolerated with no serious adverse events and no acneiform rash was detected [74]. To evaluate nimotuzumab combined with radiation alone, Crombet et al. designed a phase I/II trial of 24 previously untreated, surgically unresectable, and locally advanced SCCHN patients [31]. Only mild to moderate adverse events were reported with the antibody in combination with radiation (mucositis, fever, hypotension, and tremors) and curiously, no skin rash was apparent. Disease control rates reported in the high-dose group were 87.5% with CR rates of 56%. Additionally, it was noted that several of the patients experiencing a PR were able to become surgically resectable. Similarly, Rojo et al. designed a phase Ib trial with nimotuzumab in combination with radiation therapy in patients unable to receive chemotherapy due to poor nutritional status, cardiovascular comorbidities, or refusal to receive chemotherapy [32]. They determined that patients could tolerate nimotuzumab in combination with radiation and specifically did not experience the common acneiform rash typically associated with the inhibition of EGFR. The study was also undertaken to look for biomarkers of the EGFR/MAPK signaling pathway; nimotuzumab inhibited EGFR-phosphorylation, but had minimal effects on ERK1/2 and AKT phosphorylation. An additional randomized trial using combined nimotuzumab and radiation therapy in unresectable SCCHN patients demonstrated a median survival of 12.5 months versus 9.47 months for radiation therapy alone [33].

The benefit of nimotuzumab with radiation and chemotherapy was demonstrated by Gupta et al. who used weekly cisplatin with radiation and nimotuzumab in patients with locally advanced SCCHN [36]. The study found an objective response rate of 76% and no grade III or IV events were detected. However, without controlled data, it is difficult to ascertain the importance of this observation given the negative result of the RTOG 0522 trial. A phase IIb trial by Babu et al. evaluated inoperable SCCHN patients in treatment arms comparing nimotuzumab and chemoradiation (N + CRT) to chemoradiation (CRT) alone and nimotuzumab and radiation therapy (N + R) to radiation therapy alone (RT) [34]. Local regional control rates were higher in the arms combined with nimotuzumab. Four-year survival rates (using intent-to-treat analysis) demonstrated 47% in N + CRT versus 21% in the CRT only arm (*P* = 0.01) and 34% in N + RT versus 13 in RT only arm (not significant). Also, a biomarker study was performed on this same patient group and it was found that EGFR expression by IHC correlated with patient survival, but phosphorylated-MAPK expression did not [75]. Thus, the survival benefits seen with nimotuzumab do not appear to correlate with the inhibition of the downstream signaling molecules of EGFR and suggest alternative mechanisms of enhanced tumor cell killing.

In the palliative setting, the combination of nimotuzumab with chemotherapy (cisplatin, docetaxel, and 5-FU) in 71 locally advanced head and neck patients who were not candidates for surgery or radiation therapy was reported by Guo et al. [39]. No serious adverse reactions were noted and overall response rate was 61% (CR + PR).

*Matuzumab* (formerly EMD72000; Merck KGaA) is a humanized IgG1 monoclonal antibody targeting the EGFR receptor. An early phase I trial tested matuzumab in nine SCCHN patients and was found to have a favorable toxicity profile [76]. A second phase I trial with matuzumab confirmed a safe toxicity profile in a panel of solid tumor patients, including four head and neck patients [77]. However, no additional trials evaluating the efficacy of matuzumab alone or in combination with radiation and/or chemotherapy in SCCHN patients have been reported.

## 7. Alternative Anti-EGFR Monoclonal Antibody Summary

For palliative therapy for recurrent and/or metastatic SCCHN, the non-cetuximab monoclonal antibodies have not demonstrated significant benefit to date. However, limitations of panitumumab in the SPECTRUM trial may be as a result of the IgG2 isotype and failure to activate ADCC against the tumor. Zalutumumab also did not show a benefit, but the antibody containing treatment arm did not have a standard of care chemotherapy regimen. Nimotuzumab appears promising in combination with chemotherapy; its advantages are a humanized IgG1 which can activate ADCC but also reduces the risk of infusion reactions, treatment side effects including rash, and the development of human anti-mouse antibodies which could interfere with therapy. While these agents have demonstrated promise in Phase I and II studies, well-controlled, randomized phase III trials are necessary to demonstrate their efficacy against SCCHN.

## 8. Future Directions

The mechanism of action of EGFR targeting monoclonal antibodies is proposed to be a blockade of tumor survival signals that affect proliferation, apoptosis, and DNA repair as well as the stimulation of the immune system to recognize and target tumor cells for destruction. The specific isotype (IgG1 ≫ IgG2) of an antibody has a direct impact on its ability to activate ADCC [78]. Other antibody factors may also regulate ADCC activation. Posttranslational modifications, such as glycosylation, are important in stabilizing Fc receptor binding [79, 80]. Patel et al. demonstrated that a glycosylated form of cetuximab had a greater than four-fold increase in ADCC-mediated cell lysis compared to an aglycosylated form of cetuximab in colon cancer cell lines [81]. Towards this end, Paz-Ares et al. presented results of a Phase I study (*n* = 75) with a glycoengineered IgG1 anti-EGFR monoclonal antibody (RO5083945; also known as GA201/RG7160) and demonstrated moderate adverse events such as infusion reactions (77%), rashes (80%; 25% grade 3) and hypomagnesemia (56%). In a population of heavily pretreated metastatic solid tumor patients (including six head and neck patients), 4% had a complete or partial response, while 36% had stable disease [82]. Currently, RO5083945 is being tested in clinical trials as a single agent in head and neck cancer (NCT01046266) and in combination with chemotherapy in colon and nonsmall cell lung cancer (NCT01326000; NCT01185847).

Other modifications to EGFR monoclonal antibodies to enhance ADCC effects have also been proposed. Schlaeth et al. modified the Fc region of cetuximab to enhance NK-cell recognition and found an enhanced cytotoxicity in K-Ras mutant lung carcinoma and colon cancer cell lines [83]. Another approach was the generation of a bispecific antibody (MDX-447) directed against EGFR and the high affinity Fc receptor to enhance ADCC [84, 85]. A phase I trial demonstrated that MDX-447 was tolerated as a single agent in a population of metastatic solid tumors (*n* = 64, 23% head and neck patients); however, a treatment arm in combination with growth factor support given to enhance ADCC was not tolerated [86]. Additionally, a recent *in vitro* study found that the double modification of Fc-glycosylation and Fc-protein recognition did not significantly enhance ADCC above the effects of each individual Fc modification [87].

Other manipulations of the immune system may prove useful to enhance the effectiveness of monoclonal antibodies. Research has demonstrated the upregulation of immune receptors, such as PD-1 (inhibitory) and CD137 (stimulatory), on NK cells, T-cells, and macrophages occurs when binding to the Fc domains of monoclonal antibodies [88–90]. Thus, targeting cetuximab-induced upregulation of T-cell expressed PD-1 or CD137 with an additional antibody could enhance the ADCC contribution to tumor cell kill. Recently, it was demonstrated that treatment with an antibody blocking the PD-1 receptor resulted in objective responses of nonsmall cell lung cancer, melanoma, and renal cell carcinoma [91].

Many preclinical and clinical studies have found added benefit in targeting multiple signaling pathways with combinations of antibodies and small molecule inhibitors, but often with higher rates of toxicity. Other investigators have specifically modified monoclonal antibodies in order to target multiple signaling pathways. An antibody targeting EGFR and the insulin growth factor receptor (IGF-R1) demonstrated antitumor effects against head and neck, colon, and pancreatic tumor cell lines as well as in mouse xenograft models [92]. Another group generated a dual-targeted antibody to VEGF and EGFR. Using head and neck, lung, and colon xenografts, they found suppressed tumor growth by the dual-targeted antibody, but interestingly, to a lesser extent than the combined treatment with individual cetuximab and bevacizumab antibodies [93, 94]. Similarly, other investigators generated a dual-targeted antibody, MEHD7945A, which targets EGFR and another growth factor receptor family member, Her3. Schaefer et al. established MEHN7945A inhibited EGFR, AKT, and ERK1/2 phosphorylation *in vitro* and caused growth inhibition in a NSCLC mouse xenograft model [95]. A phase I trial evaluating the safety of MEHN7945A in humans is underway (NCT01207323).

Alternative modifications of monoclonal antibodies have included conjugation to a radioactive isotope referred to as radioimmunotherapy (RIT). In several preclinical studies, investigators found efficacy and tolerability of anti-EGFR antibodies linked to In-111, I-131, and Bi-213 (an alpha-emitting isotope) [96–98]. More recently, Liu et al. used radiolabeled Y-90 panitumumab to demonstrate a decreased proliferation of a head and neck cell line and improved survival of head and neck mouse xenografts [99]. While not in SCCHN, patients, a prior clinical trial used 188-Re labeled nimotuzumab in patients with glioblastoma multiforme and reported good tolerance to therapy [100].

One of the limitations of anti-EGFR antibody therapy is that currently, there are no biomarkers available to help clinicians predict which patients are most likely to benefit from treatment. Hitt et al. found that in recurrent/metastatic SCCHN the combination of weekly cetuximab and paclitaxel resulted in objective responses; however, no correlation between EGFR expression or copy number and overall survival was identified [62]. While it has been demonstrated that HPV-positive oropharyngeal tumors are associated with improved outcomes, it is unknown if anti-EGFR therapy is optimal for either HPV-positive or HPV-negative patients [23, 101]. Thus, additional research is needed to find biomarkers that can be reflective of antibody response as well as prognostic of outcome.

In summary, cetuximab has clearly demonstrated an advantage in both the curative and palliative treatment settings for SCCHN. Related anti-EGFR antibodies, such as nimotuzumab, have also shown efficacy, as well as a more tolerable side effect profile. Additional preclinical studies and future prospective clinical trials are necessary to find specific subsets of patients who will optimally benefit from treatment with anti-EGFR monoclonal antibodies.

## Figures and Tables

**Table 1:** Anti-EGFR monoclonal antibodies in clinical use.

Antibody	Species	IgG	Activate ADCC*	Skin reactions
Cetuximab	Chimeric human/mouse	IgG1	Y	Y
Panitumumab	Fully human	IgG2	N	Mild
Zalutumumab	Fully human	IgG1	Y	Y
Nimotuzumab	Humanized	IgG1	Y	N
Matuzumab	Humanized	IgG1	Y	Mild

*ADCC: antibody-dependent cellular cytotoxicity.

**Table 2:** Cetuximab in the curative setting.

Study	Year	Phase	Number of Patients/agent	Response (%)/Survival (months)
Cetuximab + radiation therapy				

Bonner et al. [18, 19]	2006 2010	III	*n* = 424	3 yr PFS 42 versus 31* 3 yr OS 55 versus 45* 5 yr OS 45.6 versus 36.4*

Cetuximab + chemoradiation				

Pfister et al. [20]	2006	II	*n* = 22; cisplatin	CR 13 PR 81 SD 0 DCR 94 3 yr PFS 56 3-yr OS 76
Langer et al. [21] (ECOG 3303)	2008	II	*n* = 61; platinum therapy	CR 23 PR 25 SD 31 DCR 79
Merlano et al. [22]	2011	II	*n* = 45; cisplatin	CR 71 PR 20 SD 0 DCR 94
Ang et al. [23] (RTOG 0522)	2011	III	*n* = 895; cisplatin	2 yr PFS 63 versus 64 2 yr OS 83 versus 80

CR: complete response, PR: partial response, SD: stable disease, DCR: disease control rate (CR + PR + SD), PFS: progression-free survival, and OS: overall survival; *statistically significant.

**Table 3:** Cetuximab in the palliative setting.

Study	Year	Phase	Number of Patients/agent	Response (%)/Survival (months)
Cetuximab alone				

Baselga et al. [24]	2000	I	*n* = 17	SD 69
Vermorken et al. [25]	2007	II	*n* = 103	CR 0
				PR 13
				SD 33
				DCR 46
				ORR 13

Cetuximab + chemotherapy				

Baselga et al. [26]	2005	II	*n* = 96; platinum therapy	CR 0
				PR 10
				SD 43
				DCR 53
				ORR 10
Herbst et al. [27]	2005	II	*n* = 131; cisplatin/paclitaxel or cisplatin/5-FU	CR 2
				PR 12
				SD 50
				DCR 64
				ORR 13
Burtness et al. [28]	2005	III	*n* = 117; cisplatin	ORR 26 versus 10
				PFS 4.2 versus 2.7
				OS 9.2 versus 8.0
Bourhis et al. [29]	2006	I/II	*n* = 53; platinum/5-FU	CR 4
				PR 32
				SD 38
				DCR 74
				ORR 36
Vermorken et al. [30] (EXTREME)	2008	III	*n* = 442; platinum/5-FU	ORR 36 versus 20
				DCR 81 versus 60
				PFS 5.6 versus 3.3 mo*
				OS 10.1 versus 7.4 mo*

CR: complete response, PR: partial response, SD: stable disease, DCR: disease control rate (CR + PR + SD), ORR: overall response rate, PFS: progression-free survival, and OS: overall survival; *statistically significant.

**Table 4:** Summary of alternative Anti-EGFR monoclonal antibodies.

Study	Year	Phase	Agent	Number of Patients/agent	Response (%)/Survival (months)
Locally advanced					

Antibody + radiation					

Crombet et al. [31]	2004	I/II	Nimotuzumab	*n* = 16**	CR 56
					DCR 88
					3 yr OS 67
Rojo et al. [32]	2010	I	Nimotuzumab	*n* = 10	CR 20
					PR 70
					SD 10
					DCR 90
Rodríguezet al. [33]	2010	II	Nimotuzumab	*n* = 106	CR 60
					OS 12.5 mo
Babu et al. [34]	2010	II	Nimotuzumab	*n* = 36	4 yr OS 34

Antibody + chemoradiation					

Wirth et al. [35]	2010	I	Panitumumab	*n* = 19; paclitaxel/ carboplatin	CR 69
					PR 31
					SD 0
					DCR 100
Gupta and Madholia [36]	2010	I	Nimotuzumab	*n* = 17; weekly cisplatin	CR 59
					PR 18
					SD 12
					DCR 89
Babu et al. [34]	2010	II	Nimotuzumab	*n* = 40; cisplatin	4 yr OS 47

Palliative					

Vermorken et al. [37] (SPECTRUM)	2010	III	Panitumumab	*n* = 657; platinum therapy/5-FU	ORR 36 versus 25
					PFS 5.8 versus 4.6
					OS 11.1 versus 9.0
Machiels et al. [38]	2011	III	Zalutumumab	*n* = 286; optional methotrexate	DCR 48 versus 27
					ORR 6.3 versus 1.1
					PFS 2.5 versus 2.1
					OS 6.7 versus 5.2
Guo et al. [39]	2011	I	Nimotuzumab	*n* = 71; cisplatin/docetaxel/5-FU	CR 6
					PR 55
					SD 25
					DCR 86

CR: complete response, PR: partial response, SD: stable disease, DCR: disease control rate (CR + PR + SD), ORR: overall response rate, PFS: progression-free survival, and OS: overall survival; *statistically significant; **high-dose treatment group.
